# Supplementary material for: Tuberculin skin test positivity among HIV-infected alcohol drinkers on antiretrovirals in south-western Uganda
Source: PLoS One. 2020 Jul 2;15(7):e0235261. doi: 10.1371/journal.pone.0235261 (PMC7332058; doi:10.1371/journal.pone.0235261)
Supplement: S1 File — (DOCX) [file pone.0235261.s001.docx]

### ADEPTT Study Screening Step 1

**Initial Eligibility Screening – self-report**

**DATE:** __ __ / __ __ / __ __ __ __  **ADEPTT SCREENING ID: SCT** __ __ __ __

(DAY/MONTH/YEAR)

| Completed by phone or in-person? | □ **Phone** | □ **In-person** |
| --- | --- | --- |
| Is this a Uganda ARCH prior participant? | □ **Yes** | □ **No** |
| If prior participant, enter study ID:  ADEPT Study ID: MBD __ __ __ __ BREATH Study ID: MBB __ __ __ __ | | |
| What is the patient’s sex? | □ **Male** | □ **Female** |

**Initial eligibility assessment.**

| 1. How old are you? __ __ **years 🡪** is patient 18 years or older? | □ **Yes** | □ No |
| --- | --- | --- |
| 2. Are you fluent in either Runyakole or English? | □ **Yes** | □ No |
| 3. Have you been on ART for 6 or more months? | □ **Yes** | □ No/Don’t know |
| 3a. If yes: Are you currently on or prescribed Nevirapine (NVP)? | □ Yes | □ **No** |
| 3b. If not currently on NVP: Have you taken NVP in the past 2 weeks? | □ Yes | □ **No** |
| 4. Do you live within 2 hours travel time of the ISS Clinic? | □ **Yes** | □ No |
| 5. Do you have plans to move more than 2 hours from the ISS Clinic, within the next 6 months? | □ Yes | □ **No** |
| 6. Have you ever had active TB before? | □ Yes | □ **No/Don’t know** |
| 7. Have you ever taken TB medications before, to treat or prevent TB? | □ Yes | □ **No/Don’t know** |
| 8. Are you currently taking anti-convulsion medications, or do you have any plans to take them in the future? | □ Yes | □ **No** |
| 9. Have you had any alcohol to drink in the past year? | □  **Yes** | □ **No** |
| 9a. If yes: Have you had any alcohol to drink in the past 3 months? | □ **Yes** | □ No |

Eligible: **YES** to questions 1, 2, 3 & 4; **NO/DON’T KNOW** to questions 5, 6, 7 & 8. (ticks in the unshaded boxes)

Ineligible: Currently on NVP (**YES** to 3a) or taken NVP in the past 2 weeks (**YES** to question 3b), or Prior year drinker who is not a prior 3 month drinker, but who drank 4-12 months ago (ie. **YES** to question 9 *plus* **NO** to question 9a) (ticks in a shaded box)

| **If eligible:** ask participant if they are interested in continuing with further screening.  **Eligibility status:**  □ Ineligible.  □ Eligible, declines further screening.  **(specify reason to the right)**  □ Eligible.  **(Refer to RA for consent for further screening)** | Reason for declining further screening:  □ 1 = Time barred  □ 2 = Stigma/disclosure issues  □ 3 = Needs additional approval from family member  □ 4 = Too weak  □ 5 = Not interested  □ 6 = Declines blood draw  □ 7 = Declines to answer  □ 8 = Other (specify) ________________________ |
| --- | --- |

**Notes:**

|  | Initials | Date |
| --- | --- | --- |
| QC check |  |  |
| Entry 1 |  |  |
| Entry 2 |  |  |

**Screener initials:** ____ ____
