# Supplementary material for: Tuberculin skin test positivity among HIV-infected alcohol drinkers on antiretrovirals in south-western Uganda
Source: PLoS One. 2020 Jul 2;15(7):e0235261. doi: 10.1371/journal.pone.0235261 (PMC7332058; doi:10.1371/journal.pone.0235261)
Supplement: S2 File — (DOCX) [file pone.0235261.s002.docx]

### ADEPTT Study Screening Step 1

**Initial Eligibility Screening – self-report**

**DATE:** __ __ / __ __ / __ __ __ __  **ADEPTT SCREENING ID: SCT** __ __ __ __

(DAY/MONTH/YEAR)

| Completed by phone or in-person? | □ **Phone** | □ **In-person** |
| --- | --- | --- |
| Is this a Uganda ARCH prior participant? | □ **Yes** | □ **No** |
| If prior participant, enter study ID:  ADEPT Study ID: MBD __ __ __ __ BREATH Study ID: MBB __ __ __ __ | | |
| What is the patient’s sex? | □ **Male** | □ **Female** |

**Initial eligibility assessment.**

| 1. “Oine emyaka engahi?” __ __ **years 🡪** is patient 18 years or older? | □ **Yes** | □ No |
| --- | --- | --- |
| 2. Nogambagye orunyakole nari orujungu? | □ **Yes** | □ No |
| 3. Waherize ameezi 6 nari okukiraho aha mubazi gw’okutuubya akakooko kasirimu? | □ **Yes** | □ No/Don’t know |
| 3a. If yes: Obwahati nomira Nevirapine (NVP) nari omushaho agikuhandikire? | □ Yes | □ **No** |
| 3b. Ku oraabe otakumira NVP obwahati, obiire wamizireho NVP omu sande 2 ezihingwire? | □ Yes | □ **No** |
| 4. Ahu orikutura, nobaasa kwija omu kiliniki omushaha 2 zonka? | □ **Yes** | □ No |
| 5. Oine entekateeka y’okufurukira omu mwanya ogundi aharikutwara ahaiguru yeshaha 2 kwija omu kiliniki egi omumeezi 6 agarikwija? | □ Yes | □ **No** |
| 6. Orarweireho endwara y’akakoonko? | □ Yes | □ **No/Don’t know** |
| 7. Oramizireho emibaazi y’okutangira nari y’okuragurira akakoonko enyimaho? | □ Yes | □ **No/Don’t know** |
| 8. Obwahati nomira emibaazi y’okuzibira okwesika, nari oine entekateeka y’okwija kugimira omumaisho? | □ Yes | □ **No** |
| 9. Okatungaho amarwa g’okunywa omumwaka oguhweire? | □  **Yes** | □ **No** |
| 9a. If yes: Otungireho amarwa gokunywa omumeezi 3 agahingwire? | □ **Yes** | □ No |

Eligible: **YES** to questions 1, 2, 3 & 4; **NO/DON’T KNOW** to questions 5, 6, 7 & 8. (ticks in the unshaded boxes)

Ineligible: Currently on NVP (**YES** to 3a) or taken NVP in the past 2 weeks (**YES** to question 3b), or Prior year drinker who is not a prior 3 month drinker, but who drank 4-12 months ago (ie. **YES** to question 9 *plus* **NO** to question 9a) (ticks in a shaded box)

| **If eligible:** ask participant if they are interested in continuing with further screening.  **Eligibility status:**  □ Ineligible  □ Eligible, declines further screening.  **(specify reason to the right)**  □ Eligible.  **(Refer to RA for consent for further screening)** | Reason for declining further screening:  □ 1 = Time barred  □ 2 = Stigma/disclosure issues  □ 3 = Needs additional approval from family member  □ 4 = Too weak  □ 5 = Not interested  □ 6 = Declines blood draw  □ 7 = Declines to answer  □ 8 = Other (specify) ________________________ |
| --- | --- |

**Notes:**

|  | Initials | Date |
| --- | --- | --- |
| QC check |  |  |
| Entry 1 |  |  |
| Entry 2 |  |  |

**Screener initials:** ____ ____
