# Supplementary material for: Tuberculin skin test positivity among HIV-infected alcohol drinkers on antiretrovirals in south-western Uganda
Source: PLoS One. 2020 Jul 2;15(7):e0235261. doi: 10.1371/journal.pone.0235261 (PMC7332058; doi:10.1371/journal.pone.0235261)
Supplement: S3 File — (DOCX) [file pone.0235261.s003.docx]

# ADEPTT Study Screening Step 2

**Participant Contact Sheet**

ADEPTT Screening ID: SCT __ __ __ __

ADEPTT Study ID: MBT __ __ __ __

Clinic ID: __________________________

**PRIOR TO CONTINUING, PLEASE CONFIRM:**

| 1. Did participant complete consent for further screening? | □ **Yes** | □ No |
| --- | --- | --- |
| Date of consent (dd/mm/yyyy): __ __ /__ __ / __ __ __ __ |  |  |

Continue if YES.

**PARTICIPANT INFO**

Participant Name (First name, Last name): ________________________________________

| Primary Phone Number:  ___________________________________ | Preferred time for contacting participant:  _____________________________________ |
| --- | --- |
| Alternate Phone Number:  __________________________________ | Instructions for contacting participant:  ______________________________________ |
| Address Info | ______________________________________ |
| Cell: _____________________________ | ______________________________________ |
| Parish: ___________________________ | ______________________________________ |
| Subcounty: _______________________ | Home visit comments: ___________________ |
| County: __________________________ | ______________________________________ |
| District: __________________________ | ______________________________________ |
| Landmarks: _______________________ | ______________________________________ |
| _________________________________ | ______________________________________ |
| _________________________________ | ______________________________________ |

**CONTACT PERSON INFO**

| **Contact person 1** | **Contact person 2** |
| --- | --- |
| Name: ______________________________ | Name: ________________________________ |
| Phone number: _______________________ | Phone number: ________________________ |
| Address: ____________________________  ____________________________________ | Address: ______________________________  _____________________________________ |
| Notes: ____________________________  ____________________________________  ____________________________________ | Notes: _______________________________  _____________________________________  _____________________________________ |
