# Supplementary material for: Tuberculin skin test positivity among HIV-infected alcohol drinkers on antiretrovirals in south-western Uganda
Source: PLoS One. 2020 Jul 2;15(7):e0235261. doi: 10.1371/journal.pone.0235261 (PMC7332058; doi:10.1371/journal.pone.0235261)
Supplement: S4 File — (DOCX) [file pone.0235261.s004.docx]

**ADEPTT Study Screening Step 3**

**Further eligibility screening – labs and symptoms**

**DATE:** __ __ / __ __ / __ __ __ __  **ADEPTT SCREENING ID: SCT** __ __ __ __

(DAY/MONTH/YEAR)

**PRIOR TO CONTINUING, PLEASE CONFIRM:**

| 1. Is patient eligible, per screening step 1? | □ **Yes** | □ No |
| --- | --- | --- |
| 2. Did patient consent for further screening? | □ **Yes** | □ No |
| 3. Has patient contact information been collected, per screening step 2? | □ **Yes** | □ No |

Continue if YES to questions 1-3.

**Clinical screening for TB symptoms***

| 1. Current Cough (cough within 24 hours or more) | □ Yes | □ No |
| --- | --- | --- |
| 2. Presence of fevers | □ Yes | □ No |
| 3. Presence of drenching night sweats | □ Yes | □ No |
| 4. Significant weight loss (> 10% previous body weight) | □ Yes | □ No |

*If patient has a current cough (YES to question 1 above), proceed to Screening Step 4 and evaluate for active TB NOW. Return to this form if patient is confirmed clear of active TB.

*If patient has any symptoms (YES to questions 2-4 above), but does NOT have a current cough (NO to question 1 above), continue with this form. Then, continue to Screening Step 4 for further evaluation for active TB.

**Pregnancy****

| If female, ask the patient:  “Are you currently pregnant?” | □ Yes | □ No/Don’t know | □ Not applicable (male, or female >60 years old) |
| --- | --- | --- | --- |

**If a woman knows that she is pregnant (replies yes above), jump to eligibility status box (Group 6, Ineligible). Do not conduct urine testing or LFT testing.

**If a woman replies no/don’t know, continue with this form. Collect urine for pregnancy test.

**If not applicable, continue with this form.

**Urine Pregnancy Result:^β^**

| □ Positive | □ Negative | □ Not done |
| --- | --- | --- |

^β^ If patient is positive for pregnancy, jump to eligibility status box (Group 6, Ineligible). Do not test LFTs.

**SCT** __ __ __ __

**Liver Function Tests (only for those who are not pregnant)**

| **Liver test** | **Value (IU/L)** | **ULN* for test** | **High?* (>2x ULN)** | | **Not done** |
| --- | --- | --- | --- | --- | --- |
| **AST** | __ __ __ | __ __ __ | □ Yes | □ No | □ |
| **ALT** | __ __ __ | __ __ __ | □ Yes | □ No | □ |
| Eligible: **NO** high liver function tests | | *ULN = upper limit of normal | |  |  |

**Determine and select participant status, using info above:**

|  | **Eligibility group** | **Pregnancy**  **(women only)** | **ALT or**  **AST*** | **Symptoms** |  | **Next step** |
| --- | --- | --- | --- | --- | --- | --- |
| □ | 1 | Not pregnant | ≤2x ULN | No symptoms |  | Place PPD |
| □ | 2 |  | ≤2x ULN | >0 symptoms, no cough |  | Place PPD, go to Screening Step 4 (further evaluation for active TB) |
| □ | 3 |  | ≤2x ULN | Any cough |  | Place PPD *ONLY* ***AFTER*** *CONFIRMED CLEAR OF ACTIVE TB (Screening Step 4)*. |
| □ | 4 |  | >2x ULN | 0 symptoms |  | Exclude, refer to ISS Clinic TB Infection/screening focal person |
| □ | 5 |  | >2x ULN | 1-4 symptoms |  | Exclude, refer to ISS Clinic TB Infection/screening focal person |
| □ | 6 | Pregnant | Not done | 0-4 symptoms |  | Exclude |

**Place PPD for TST (all eligible patients: group 1, 2, or 3)**

| **PPD placed?** | **Date placed** | **Time placed** | **Placement** | **Lot/Batch #** | **Expiration date** |
| --- | --- | --- | --- | --- | --- |
| □ Yes  □ No | __ __ / __ __ / __ __ __ __  (DAY/MONTH/YEAR) | __ __:__ __  □ AM □ PM | □ Left Forearm  □ Right Forearm |  | __ __ / __ __ / __ __ __ __  (DAY/MONTH/YEAR) |

**Notes:**

**RA Initials:** __ __ **Signature:** ……………………………………………………………..

|  | Initials | Date |
| --- | --- | --- |
| QC check |  |  |
| Entry 1 |  |  |
| Entry 2 |  |  |
