# Supplementary material for: Tuberculin skin test positivity among HIV-infected alcohol drinkers on antiretrovirals in south-western Uganda
Source: PLoS One. 2020 Jul 2;15(7):e0235261. doi: 10.1371/journal.pone.0235261 (PMC7332058; doi:10.1371/journal.pone.0235261)
Supplement: S5 File — (DOCX) [file pone.0235261.s005.docx]

**ADEPTT Study Screening Step 4**

**Further Eligibility Screening - Chest X-Ray and TB Laboratory Form**

**DATE:** __ __ / __ __ / __ __ __ __ **ADEPTT SCREENING ID: SCT** __ __ __ __

(DAY/MONTH/YEAR)

**PRIOR TO CONTINUING, PLEASE CONFIRM:**

| 1. Did the participant report any clinical TB symptoms in Screening Step 3 (cough, fevers, night sweats, weight loss)? | □ **Yes** | □ No |
| --- | --- | --- |

Continue if YES. Do NOT continue if no symptoms were reported in step 3.

**CHEST X-RAY:**

**If female:** confirm that participant is not pregnant. If participant already had a urine test as part of Step 3, refer to those results. If participant has not had a urine pregnancy test yet as part of screening, conduct pregnancy test.

**If pregnant**: do **NOT** conduct chest x-ray.

| **Pregnancy test result:** | □ **Not pregnant** | □ Pregnant | □ **N/A (male participant)** |
| --- | --- | --- | --- |

| Chest x-ray ordered and request form filled by: _____________________________________ | | |
| --- | --- | --- |
| Chest x-ray first read by: ____________________________________ | | |
| Chest x-ray confirmed by: ___________________________________ | | |
| **Final disposition:** | □ CXRAY suggestive of pulmonary TB | □ CXRAY not suggestive of TB |

**SPUTUM SAMPLE(S)*:**

|  |  |  |
| --- | --- | --- |
| **SAMPLE 1. Sample date**: __ __ / __ __ / __ __ __ __  (DAY/MONTH/YEAR) | **Sample type:** | □ Early morning sample  □ Spot  □ Not able to produce sample |
| **Date of GenXpert test:** __ __ / __ __ / __ __ __ __  (DAY/MONTH/YEAR) |  |  |
|  |  |  |
| **Results:** □ MTB detected □ MTB not detected  □ Inconclusive □ Not done | **If MTB detected:** | □ Rifampicin sensitive  □ Rifampicin resistant |

| ***ONLY COLLECT A SECOND SPUTUM SAMPLE IF SAMPLE 1 IS INCONCLUSIVE/INSUFFICIENT.** | | |
| --- | --- | --- |
| **SAMPLE 2*. Sample date**: __ __ / __ __ / __ __ __ __  (DAY/MONTH/YEAR) | **Sample type:** | □ Early morning sample  □ Spot  □ Not able to produce sample |
| **Date of GenXpert test:** __ __ / __ __ / __ __ __ __  (DAY/MONTH/YEAR) |  |  |
|  |  |  |
| **Results:** □ MTB detected □ MTB not detected  □ Inconclusive □ Not done | **If MTB detected:** | □ Rifampicin sensitive  □ Rifampicin resistant |

| **SUMMARY: Is patient confirmed to be clear of active TB?** | □ **Yes** | □ No |
| --- | --- | --- |

Eligible: **YES**, confirmed to be clear of TB.

**Notes:**

|  | Initials | Date |
| --- | --- | --- |
| QC check |  |  |
| Entry 1 |  |  |
| Entry 2 |  |  |

**RA Initials:** __ __ **Signature:** ……………………………………………………………..
