# Supplementary material for: Tuberculin skin test positivity among HIV-infected alcohol drinkers on antiretrovirals in south-western Uganda
Source: PLoS One. 2020 Jul 2;15(7):e0235261. doi: 10.1371/journal.pone.0235261 (PMC7332058; doi:10.1371/journal.pone.0235261)
Supplement: S6 File — (DOCX) [file pone.0235261.s006.docx]

**ADEPTT Study Screening Step 5**

**Final screening eligibility - TST**

**DATE:** __ __ / __ __ / __ __ __ __  **ADEPTT SCREENING ID: SCT** __ __ __ __

(DAY/MONTH/YEAR)

**PRIOR TO CONTINUING, PLEASE CONFIRM:**

| 1. Is the participant confirmed to be clear of active TB? | □ **Yes** | □ No |
| --- | --- | --- |

Continue if YES

**TUBERCULIN SKIN TEST (TST)**

| **Date TST read** | **Time TST read** | **Hours since PPD placed*** | **Patient induration in millimeters (mm)** | **Positive**  **≥ 5 mm** | **Negative**  **<5 mm** | **Not read** | **Other description** |
| --- | --- | --- | --- | --- | --- | --- | --- |
| __ __ / __ __ / __ __ __ __  (DAY/MONTH/YEAR) | __ __:__ __ □ AM □ PM | __ __ | __ __ | □ | □ | □ |  |

*If less than 48 hours or more than 72 hours have passed since PPD was placed: still read and record results; refer to protocol.

Eligible: **POSITIVE results (≥5 mm)**

**If eligible:** Invite participant for main study consent.

| **Final ADEPTT eligibility:** | Reason for declining enrollment  □ 1 = Time barred  □ 2 = Stigma/disclosure issues  □ 3 = Needs additional approval from family member  □ 4 = Too weak  □ 5 = Not interested  □ 6 = Declines blood draw  □ 7 = Declines to answer  □ 8 = Other (specify) ________________________ |
| --- | --- |
| □ Ineligible. |  |
| □ Eligible, declines consent.  **(specify reason to right)** |  |
| □ Eligible.  **(continue and complete study consent next)** |  |

**Study Consent (*if eligible*):**

| Was consent to participate in the full study obtained? | □ **Yes** | □ No |
| --- | --- | --- |
| Date of consent (dd/mm/yyyy): __ __ /__ __ / __ __ __ __ | | |

**After consent has been obtained, and participant enrolled: Assign participant study ID. MBT __ __ __ __**

**Notes:**

|  | Initials | Date |
| --- | --- | --- |
| QC check |  |  |
| Entry 1 |  |  |
| Entry 2 |  |  |

**RA Initials:** __ __ **Signature:** ……………………………………………………..
