# Supplementary material for: Tuberculin skin test positivity among HIV-infected alcohol drinkers on antiretrovirals in south-western Uganda
Source: PLoS One. 2020 Jul 2;15(7):e0235261. doi: 10.1371/journal.pone.0235261 (PMC7332058; doi:10.1371/journal.pone.0235261)
Supplement: Attachment — Submitted filename: Response to Reviewers June 06 2020.doc [file pone.0235261.s009.doc]

Dr. Muyindike Winnie Rhoda

Mbarara University of Science and Technology

& Mbarara Regional Referral Hospital

P.O. Box 1410, Mbarara, Uganda

June 06, 2020

The Academic Editor

PLOS ONE

Dear Editor,

**Re: Responses to Manuscript PONE-D-19-35988R1: “Tuberculin skin test positivity among HIV-infected alcohol drinkers on antiretrovirals in south-western Uganda”**

Thank you for the provisional acceptance for our manuscript, the valuable time you spent reviewing our previously submitted manuscripts and the second set of suggestions given to us. We also appreciate the opportunity to respond to the editor’s and reviewer’s recommendations. Below, please find a summary of the editor and reviewer’s comments and our responses to indicate how each point is addressed in the revised manuscript.

**Review Comments to the Author**

Responses specific to Reviewer #1’s comments:

Comment to question, “Have the authors made all data underlying the findings in their manuscript fully available? reviewer 1 said No:

Response: We have prepared an anonymized dataset for all the 418 participants screened prior to conducting the tuberculin skin test (TST). The dataset that is attached on the 2^nd^ resubmission includes those included for detailed analysis (295) and those excluded from the detailed analysis (123) who didn’t meet the criteria for inclusion for detailed analysis. This will be submitted as a Supporting Information file.

Responses specific to Reviewer #2’s comments:

Line 31: Comments have been addressed, but I would still add “….current evidence…”

Response: We have added the word ‘current’ in the suggested position in line 31

Line 131-136: Comments have been addressed, but phrasing is still in discussion form, maybe best to some of it in the discussion as a limitation of this work. (line 131) “TST may not be a perfect test for LTBI but it is widely accepted as a reasonable proxy [28] and”
and (line 134) “Both tests perform reasonably well in high-TB burden settings and correlate well with proxy measures of exposure [28].” I would move back to discussion and expand.

Response: We have moved the suggested parts back in the limitations paragraph of the discussion and expanded. The previous line 131 and 134 now appear in the limitations under discussion, lines 292 to 299.

I would also move the sentences around in the method section (line 134/5) “TST may have reduced sensitivity in immune-compromised individuals such as… “ before the sentence starting (line 130) “A TST…”

Response: We have moved the suggested sentences around in the methods section. The previous lines 134/5 is now line 131. It has been inserted before the previous sentence which described the 5mm reading for a positive TST which is now line 132.

281-285 comments have been addressed, but I would leave out (line 286) “However, the exclusions do not introduce a systematic bias that we see.”

Response: We have deleted the line that previously read, “ However, the exclusions do not introduce a systematic bias that we see.”

Thank you again for the consideration to process our manuscript for publication. We hope that with these revisions, the manuscript is now suitable for publication in PLOS ONE.

Submitted by;

Dr Winnie R Muyindike

Mbarara University of Science and Technology/Referral Hospital, Mbarara, Uganda

Email: [wmuyindike@gmail.com](mailto:wmuyindike@gmail.com)

Phone: +256 772 521619
